# The Complex Nature of Hippocampal-Striatal Interactions in Spatial Navigation

**DOI:** 10.3389/fnhum.2018.00250

**Published:** 2018-06-21

**Authors:** Sarah C. Goodroe, Jon Starnes, Thackery I. Brown

**Affiliations:** School of Psychology, Georgia Institute of Technology, Atlanta, GA, United States

**Keywords:** hippocampus, caudate, striatum, navigation, planning, strategies

## Abstract

Decades of research have established the importance of the hippocampus for episodic and spatial memory. In spatial navigation tasks, the role of the hippocampus has been classically juxtaposed with the role of the dorsal striatum, the latter of which has been characterized as a system important for implementing stimulus-response and action-outcome associations. In many neuroimaging paradigms, this has been explored through contrasting way finding and route-following behavior. The distinction between the contributions of the hippocampus and striatum to spatial navigation has been supported by extensive literature. Convergent research has also underscored the fact that these different memory systems can interact in dynamic ways and contribute to a broad range of navigational scenarios. For example, although familiar routes may often be navigable based on stimulus-response associations, hippocampal episodic memory mechanisms can also contribute to egocentric route-oriented memory, enabling recall of context-dependent sequences of landmarks or the actions to be made at decision points. Additionally, the literature has stressed the importance of *subdividing* the striatum into functional gradients—with more ventral and medial components being important for the behavioral expression of hippocampal-dependent spatial memories. More research is needed to reveal how networks involving these regions process and respond to dynamic changes in memory and control demands over the course of navigational events. In this Perspective article, we suggest that a critical direction for navigation research is to further characterize how hippocampal and striatal subdivisions interact in different navigational contexts.

## Introduction

**Figure 1:**
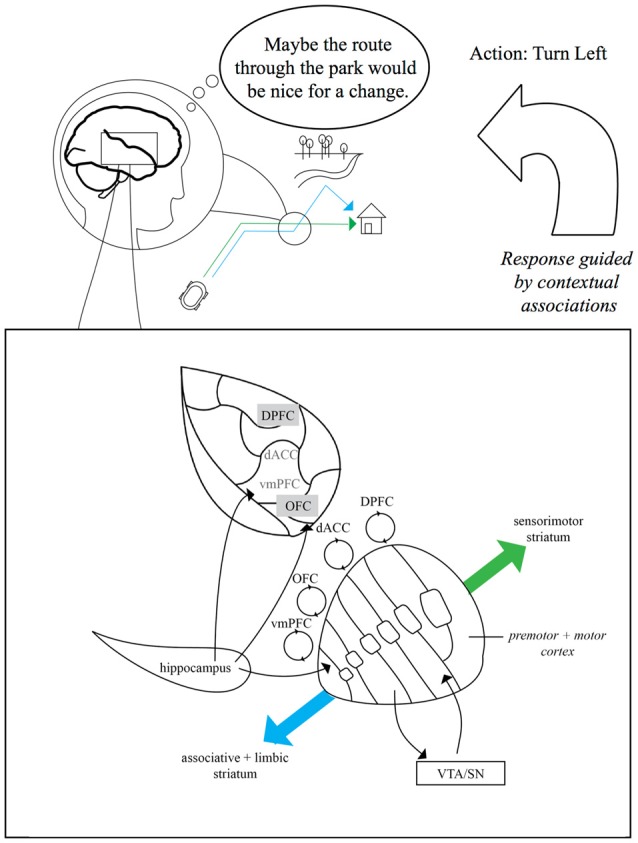
Schematic of striatal anatomy and hippocampal-dorsal striatal contributions to navigational route flexibility. Inset box: coronal view of the striatum and some relevant connections. The striatum receives dopaminergic input from ventral tegmental area (VTA) and the substantia nigra (SN). Primate dorsomedial striatum (DMS) is characterized by partially-overlapping reciprocal connectivity with ventral striatum (VS) and ventromedial prefrontal cortex (PFC) (vmPFC), orbitofrontal cortex (OFC), dorsal anterior cingulate cortex (dACC) and dorsal prefrontal areas (DPFC). Striatum-PFC connectivity is a continuum that changes as we move dorsal and lateral from “limbic” and “associative” striatum (DMS) towards the “sensorimotor” striatum (DLS). DMS function can interface with hippocampal function via hippocampal inputs to VS and to shared prefrontal targets (particularly vmPFC and OFC). Upper figure: conceptual example of the balance between model-free and model-based navigation. Model-free navigation (e.g., green “habitual” route) may be governed by DLS. When goals change (e.g., blue alternative route), mnemonic input from the hippocampus to VS and PFC enables contextual traces (e.g., goal states and event memory cues) to guide model-based action selection and updating via DMS-frontrostriatal loops. In this example, the navigator might disengage from an overlearned route home (green route), suppressing a “straight” action in favor of a goal-directed left-turn into the park (blue route). Gray text boxes on PFC indicate subdivisions on the lateral surface.

In our daily lives, we are continually faced with decisions about where to go next and how to get there. Making these decisions can rely on a map-like representation of the overall spatial environment which we occupy, as well as retrieval of memories for routes that connect different locations. Flexible selection between learned routes to our destinations often involves disambiguating memory traces for similar, or even physically overlapping, locations. Alternative routes can introduce computational demands on declarative memory and response selection circuitry, which can vary depending on how ambiguous the current context is and how well-learned the behaviors are. As we navigate branches between overlapping routes (Figure 1) or attempt to retrieve different memories of the same location, we may need to rely on neural systems that: (a) enable behavioral flexibility and cognitive control; and (b) enable context-dependent retrieval of episodes. The striatum and hippocampus, respectively, are parts of these systems, and are functionally linked via the prefrontal cortex (PFC). This Perspective article, highlights research indicating that the functions of these structures may interact to enable the types of flexible navigational decisions we often make in our daily lives.

Our perspective article is focused on two overlapping literatures, which we briefly survey below and then elaborate on in separate sections. Building on the famous discovery of “place cells” and on landmark case studies involving patient H.M., research has established the importance of the hippocampus for both spatial and episodic memory (Scoville and Milner, 1957; O’Keefe and Dostrovsky, 1971; O’Keefe and Nadel, 1978; O’Keefe et al., 1998; Eichenbaum et al., 2007; Squire et al., 2007; Harand et al., 2012; Corkin, 2013; Squire and Dede, 2015). Critically, space is a core component within the definition of episodic memory (Tulving, 1972). Findings in rodents and humans: (a) demonstrate context-dependent coding of space in the hippocampus; and (b) indicate that the hippocampus is important for disambiguation and episodic retrieval of overlapping navigational memories (Wood et al., 2000; Ferbinteanu and Shapiro, 2003; Lee et al., 2006; Smith and Mizumori, 2006; Brown et al., 2010, 2012; Brown and Stern, 2014; Brown et al., 2014; Chanales et al., 2017).

Navigational route disambiguation provides an important example of the link between navigation and episodic memory mechanisms (Hasselmo and Eichenbaum, 2005), because seminal fMRI studies have emphasized preferential engagement of the hippocampus for map-based over egocentric landmark or route-based navigation (Hartley et al., 2003; Iaria et al., 2003; Doeller et al., 2008; Marchette et al., 2011). Such work has been critical for establishing functional links between the human hippocampus and “map-like” representations of environments that could be supported by place cells observed in rodents and recently humans (O’Keefe and Nadel, 1978; Thompson and Best, 1989; O’Keefe et al., 1998; Eichenbaum et al., 1999; Eichenbaum, 2000; Ekstrom et al., 2003). However, retrieval of navigational routes can be framed as retrieval of sequential, spatio-temporal events that may draw upon episodic memory mechanisms (particularly when overlap between routes increases contextual-dependency of behavior).

Indeed, the rodent literature demonstrates that place cells fire in sequences and along routes, potentially helping link specific sequences of turns and landmarks to eventual rewards and goals (Johnson and Redish, 2007; Wikenheiser and Redish, 2015). Therefore, particularly in cases when stimulus-response associations may be inadequate for overcoming multiple possible actions for a location, hippocampal-dependent memory for sequences can enable accurate goal-directed behavior. Paralleling data from neural recordings in rodents (Wikenheiser and Redish, 2015), we have recently demonstrated evidence in humans for the hippocampus supporting such a retrieval mechanism in a highly familiar environment (Brown et al., 2016). These data illustrate the broader point, which we revisit in the next section, that in some circumstances the medial temporal lobe (MTL) declarative memory system may cooperate with components of the striatum to retrieve memories (Scimeca and Badre, 2012) and navigate decision points along routes (Johnson et al., 2007).

Our Perspective article also focuses on the role of the dorsal striatum in navigation. As noted above, a classic distinction has been made between the functioning of the dorsal striatum and the hippocampus (Packard and McGaugh, 1996; Hartley et al., 2003; Iaria et al., 2003; Doeller et al., 2008; Marchette et al., 2011). Importantly, it is well-known that the dorsal striatum is not a functionally-uniform region. It can be *subdivided* into functional gradients, with more ventral and medial components being important for the behavioral acquisition and expression of hippocampal-dependent memories and alternate behaviors in maze environments (Devan and White, 1999; Ragozzino, 2002; DeCoteau et al., 2007; Thorn et al., 2010). Indeed, recent research in both humans and rodents has emphasized that although one function of the striatum is the formation and execution of inflexible stimulus-response associations (“habits”; Yin and Knowlton, 2006), frontostriatal loops may also enable us to flexibly update prepotent navigational responses and leverage mnemonic signals from the hippocampus to drive context-dependent spatial decision-making and responding (Brown et al., 2012; Brown and Stern, 2014; Ferbinteanu, 2016; Figure 1).

The distinct hippocampal and dorsal striatal systems may dynamically interact to enable us to fluidly transition between more rigid and flexible navigational behaviors, and to translate declarative memory into guidance of ongoing actions. The literature suggests these interactions between dorsal striatum and hippocampal regions may be mediated by PFC and ventral striatum (VS).

## Hippocampal Mechanisms for Spatial Mapping and Route Learning

As noted above, much imaging research has focused on the role of the hippocampus in retrieving “map-like” declarative knowledge of spatial environments. Such hippocampal-dependent knowledge is putatively built upon underlying spatial mapping mechanisms in the MTL (place and grid cells, Buzsáki and Moser, 2013) and the broader network supporting allocentric reference frames in navigation (Ekstrom et al., 2014). In some conceptual frameworks, hippocampal function has been explicitly linked with path integration (Wolbers et al., 2007; Sherrill et al., 2013; Chrastil et al., 2015). By contrast, egocentric landmark or route-oriented navigation has been attributed to striatal-dependent motor associations for environmental cues (Hartley et al., 2003; Iaria et al., 2003; Doeller et al., 2008; Marchette et al., 2011).

In a recent critical review of the literature, Ekstrom et al. (2014) argued that the study of the cognitive basis of allocentric memory has been complicated by the fact that many imaging studies may involve a blending of allocentric and egocentric representations in some form. Consequently, they argue that attributing signals to one reference frame or the other may be challenging (for broader discussion, see also Wolbers and Wiener, 2014). Indeed, the same hippocampal cell can encode place information when an animal engages in place-based strategies, and sequential state information during route-based navigation (Cabral et al., 2014). Ekstrom et al. (2014) argue that allocentric memory need not emerge from a singular type of representation in one region (such as the hippocampus), but could arise from a convergence of partially-overlapping computations in a broad network of areas that have been attributed to either allocentric and egocentric reference frames in various experiments. Here, we emphasize complementary evidence linking hippocampal function to route-based navigation. The rodent hippocampus has been explicitly linked to egocentric route navigation by demonstrating that mice lacking CA1 NMDA receptors were impaired in acquisition of both egocentric and allocentric memory for navigation (Rondi-Reig et al., 2006). This group has recently extended this to humans, with evidence the hippocampal association with route-based navigation may be left lateralized (Iglói et al., 2010).

One bridge between allocentric and egocentric navigational memory is to consider hippocampal representations of location as a mechanism underlying the ability to associate stimuli and experiences across space and time (Eichenbaum and Cohen, 2014). In the real-world, episodic memories encompass the “who, what, when and where” of an experience and, thus, require the ability to embed non-spatial information (e.g., faces and objects) in memory for environments (e.g., Burgess et al., 2001; reviewed in Burgess et al., 2002; Bird and Burgess, 2008). The early discovery of “place cells” in the hippocampus (O’Keefe and Dostrovsky, 1971; O’Keefe, 1976) lent critical neurobiological support to the concept of a “cognitive map” (Tolman, 1948; O’Keefe and Nadel, 1978). Since then this spatial mapping framework has been extended by evidence that place cell activity during route navigation exhibits hallmarks of episodic memory: the ability to fire in sequences, and in a context-dependent manner (Wood et al., 2000; Ferbinteanu and Shapiro, 2003; Lee et al., 2006; Smith and Mizumori, 2006; Johnson and Redish, 2007; Wikenheiser and Redish, 2015; Ólafsdóttir et al., 2015). Although relatively sparse, complementary work in humans has shown that hippocampal gray matter volume and function similarly support context-dependent route navigation (Brown et al., 2010, 2012, 2014; Brown and Stern, 2014; Chanales et al., 2017). The role of the human hippocampus in route-based navigation (Iglói et al., 2010) includes specific computations relevant to goal-directed decision-making, such as encoding path length of routes to goals (Howard et al., 2014).

Indeed, it is important to note that access to a spatial representation does not necessarily lead to a map-based strategy, as in the case of place-recognition triggered response strategies (Trullier et al., 1997). It is also important to consider how sequential firing for navigational routes could facilitate mechanisms of memory formation, retrieval, and even planning. Sequential firing potentially helps link specific turns and landmarks to memories for specific events and to eventual rewards and goals, and continued experience with routes may ultimately give rise to more “semanticized” map-level representations (Buzsáki, 2005; Buzsáki and Moser, 2013). During rest periods, sequential place cell firing could reflect the hippocampus “practicing” encoded route memories in service of consolidation—enabling long-term spatial memory (Wilson and McNaughton, 1994; McKenzie and Eichenbaum, 2011). Indeed, replay following new spatial learning predicts subsequent memory performance (Dupret et al., 2010) and post-encoding disruption of hippocampal sharp-wave ripples impairs subsequent spatial memory (Girardeau et al., 2009). Likewise, triggering place cell activity during sleep influences waking spatial behavior (De Lavilléon et al., 2015).

Similarly, sequential place expression may give rise to mnemonic signals important for *planning* routes (Ólafsdóttir et al., 2018). Specifically, place coding associated with cognitive mapping can also contribute to order sequencing of goal-oriented spatio-behavioral events (Howard et al., 2014). This includes prospective replay of navigational routes in both rodents and humans (Johnson and Redish, 2007; Foster and Knierim, 2012; Wikenheiser and Redish, 2015; Brown et al., 2016), as well as coding of goal and path distances (Sherrill et al., 2013; Howard et al., 2014; Spiers et al., 2017). Such evidence from rodent and human spatial navigation literature is suggestive of mechanistic links between hippocampal memory and prospective planning and motor selection processes.

These scenarios present interesting opportunities for future research. For example, overlapping routes present situations in which the brain may dynamically shift between stimulus-response and hippocampal-dependent associations as the navigator traverses more and less automated decision points. This shift may be associated with engagement of prefrontal cognitive control and evaluation processes (Brown et al., 2012). How these dynamics are mediated, and how the brain detects a need for high control in some states vs. an opportunity to release cognitive resources in others (e.g., when behavior can return to a more habitual state), is an area ripe for additional research (we return to this idea in the next section). There is also a need for research testing models of how route navigation can be solved by either: (a) an initial retrieval of the sequence that is maintained in working memory until critical decision points (Zilli and Hasselmo, 2008a); or (b) retrieval of necessary information for critical decisions that are cued at the decision points themselves (Zilli and Hasselmo, 2008b). What network dynamics determine when and how route-oriented navigation is guided by prospective or retrospective hippocampal processes?

One takeaway from the above literature is that a distinction between hippocampal and striatal function in navigation may be better framed according to the computational process, rather than the type of information (e.g., place-oriented). In particular some researchers have advocated characterizing navigation within a reinforcement learning perspective of behavior (for review and recent fMRI work, see Khamassi and Humphries, 2012; Simon and Daw, 2011), with the contributions of the hippocampus and different striatal subdivisions attributed to model-based or model-free mechanisms. As framed by this line of work, the hippocampus (and components of the striatum discussed further in the “Striatal Subdivisions and the Translation of Memory Into Behavior” section) may contribute to goal-directed, model-based behavior. Independent of perspective (allocentric or egocentric) or information type (explicitly spatial or not), this circuitry is theorized to enable construction and updating of a world/task model.

One important point is that despite its central role in spatial navigation, the hippocampus is not anatomically positioned to directly control motor behavior. However, hippocampal regions have direct connections with PFC and frontostriatal loops (by proxy) (Alexander et al., 1986; Cavada et al., 2000; Middleton and Strick, 2002; Haber et al., 2006; Roberts et al., 2007; Figure 1). Moreover, the hippocampus sends direct projections to the VS (Thierry et al., 2000), which can provide an explicit link between hippocampal memory output and striatal reward signals that strongly influence goal-directed behavior (Khamassi and Humphries, 2012). These hippocampal-prefrontal-striatal connections could enable flexible decision-making and behavioral updating (Brown et al., 2012, 2016; Brown and Stern, 2014; Ferbinteanu, 2016) based on the goal-directed output from the hippocampus during planning and navigation. Models of navigation have proposed that spatially-diffuse firing of place cells in the subiculum of the hippocampus may support coding of goals (Burgess and O’Keefe, 1996). Such signals could reflect interactions with PFC and reward circuitry. Babayan et al. (2017) showed that the hippocampus, VS and dorsomedial striatum (DMS) operate as a network in service of route navigation. In their study, the hippocampus served as network node involved in learning a sequential egocentric strategy, and as a network hub when sustaining sequence-based navigation. Therefore, although hippocampal and striatal forms of memory may differ in fundamental ways (White and McDonald, 2002; Graybiel and Grafton, 2015), our ability to engage in model-based navigation in real-world settings may draw on subdivisions of both regions. More generally, integration of MTL and frontostriatal computations may be important for memory and memory-guided behavior in many scenarios as a function of their combined relevance to current task demands (Moses et al., 2010; Ben-Yakov and Dudai, 2011; Ross et al., 2011; Sadeh et al., 2011). Below, we further outline the potential complementary roles of dorsal striatal in real-life navigational contexts.

## Striatal Subdivisions and the Translation of Memory Into Behavior

The striatum is a large, heterogeneous region of the brain that can broadly be divided into ventral, dorsomedial and dorsolateral subregions, although this organization may be best viewed as a gradient of anatomical connections with different prefrontal divisions and with a different functional emphasis within regions (Haber and Knutson, 2010). The striatum is a principal interface in the motor/reward/addiction circuit that receives glutamatergic inputs from the amygdala, thalamus, hippocampus and cortex; and dopaminergic inputs from the ventral tegmental area (VTA) and the substantia nigra (SN; Haber and Knutson, 2010; Yager et al., 2015). Although the striatum is a component of the “reward” circuit, it also interacts with memory, emotion, and cognitive planning areas of the MTL and PFC to contribute flexibility to responses and decision making (Haber and Knutson, 2010; Brown et al., 2012; Scimeca and Badre, 2012; Yager et al., 2015; Ferbinteanu, 2016).

In spatial navigation research, a classic dichotomy between hippocampal-dependent and dorsal striatal-dependent memory emerged with Packard and McGaugh’s (1996) demonstration that hippocampal function supported a “navigate-to-place” strategy, as opposed to a dorsal striatum-dependent response-learning strategy. In this classic experiment, inactivation of the hippocampus resulted in a blockade of place learning, whereas inactivation of the caudate (a component of the dorsal striatum) resulted in a blockade of response learning. As discussed above, this classic dissociation has found extensive parallels in human navigation research (Hartley et al., 2003; Iaria et al., 2003; Doeller et al., 2008; Marchette et al., 2011). Indeed, gray matter volume in the hippocampus and caudate nucleus differentially correlate with the predisposition of a person to rely on spatial knowledge or response-based strategies to solve navigational problems (Bohbot et al., 2007; Konishi and Bohbot, 2013).

Early work in rodents (Devan and White, 1999) also prompted attention to functional subdivisions of the striatum. Devan and White’s (1999) findings indicated that DMS, in contrast to dorsolateral striatum (DLS), might be important for promoting flexible, hippocampally-dependent navigation behavior. They demonstrated that lesions to medial caudate-putamen resulted in a preference for cue-guided responses, whereas lesions to the lateral caudate-putamen resulted in a preference for spatial responses. This finding contributed to the emergent idea that through parallel pathways, which can interact via the hippocampal-prefrontal-striatal connectivity described above (Figure 1), mnemonic signals and decision-making processes could regulate action selection and “downstream” processing in DLS and motor cortex (Brown et al., 2012).

Adopting a reinforcement-learning perspective of model-based and model-free navigation may be advantageous over attributing striatal function to response vs. place strategies (Simon and Daw, 2011; Khamassi and Humphries, 2012). Specifically, in conjunction with the hippocampus, the DMS may support behavior based on an inner representation of world or task space and model-based processing, whereas model-free response learning can underlie “habits” and may be attributable to the DLS (Daw et al., 2005; Khamassi and Humphries, 2012). The tightly reward-related VS may play a key role in the model building process for model-based action. Model-based control is predictive, based on action-outcome contingencies that can quickly incorporate changes in goal-relevant information (reward) throughout a world model. This gives rise to a system that can support goal-directed changes in behavior and contrasts with model-free responses emerging from gradually-learned independent action-state representations (Simon and Daw, 2011; Khamassi and Humphries, 2012). taxonomy of striatal subdivision mechanisms offers a view of how this system enables transitions from flexible to relatively automated navigational behavior, without attributing a specific information type to simple state-response information (model-free) or predictive action-outcome based processing (model-based).

One aspect of our view is that the dorsal striatum functions as a prepotent motor response regulating structure. That is, the striatum collectively enables habitual motor control, but increasingly medial and ventral components interface with dorsolateral, medial and ventral/orbital PFC to help govern *flexible* suppression, selection and updating of responses (Yin and Knowlton, 2004, 2006; Haber et al., 2006; Haber and Knutson, 2010). Through a pattern of partially-overlapping reciprocal connections (Figure 1), reward and goal-oriented processing in PFC and its associated VS and DMS subdivisions can exert control over behavior that could otherwise be governed by response associations. Having a “habit” system is very adaptive, and its utility can be exemplified by navigational scenarios in which responses based on stimulus-response associations can free up cognition for, e.g., holding conversations, monitoring for threats (Schwabe and Wolf, 2013), or planning how to achieve unrelated goals. However, the functional continuum in the dorsal striatum, grounded in differential connectivity with prefrontal subdivisions (Yin and Knowlton, 2006; Haber and Knutson, 2010) gives rise to an elegant system that can also re-engage with such ongoing behavior to exert cognitive control over, or update, our motor response program when it’s adaptive to do so. Studies targeting how this system can learn and implement stimulus-control state associations in spatial environments will be of substantial impact for theories of when and how navigational behaviors are executed in more or less automated manners. It has been proposed that this depends on function of the caudate and its interactions with the hippocampus in humans (Jiang et al., 2015; Chiu et al., 2017).

Although surprisingly under-studied in human navigation, the view that the caudate is involved in flexible behavior is not novel. Research has long associated striatal function with set-shifting, cognitive flexibility, and rule learning (Alexander et al., 1986; Middleton and Strick, 2000; Seger and Cincotta, 2005; Graham et al., 2009; Vaghi et al., 2017). One extension of the human navigation literature addressing this point (e.g., Brown et al., 2012) is evidence that indirect connectivity between the hippocampus and caudate may enable these distinct memory systems to compensate for one another and preserve navigation ability when one system starts to fail. This has been observed with hippocampal compensation for route-based navigation in patients with Huntington’s Disease (Voermans et al., 2004).

In keeping with Khamassi and Humphries’s (2012) theoretical perspective, the DMS contributes to non-habitual route-based navigation in part through interactions with hippocampus. The DMS supports the ability of rodents to learn and execute alternative behaviors in environments (Ragozzino, 2002; DeCoteau et al., 2007; Ragozzino et al., 2009; Baker and Ragozzino, 2014). Specifically, the DMS is a key network node alongside the VS and hippocampus for egocentric route-based navigation (Babayan et al., 2017), and DeCoteau et al. (2007) demonstrated that hippocampal and DMS theta oscillations are tightly coupled during critical choice periods in T-mazes. Such functional connectivity data indicate that these two systems actively interact in service of goal-directed route navigation. This work has been mirrored in recent fMRI research in humans (Brown et al., 2010, 2012), and has revealed parallel, dynamic changes in the hippocampus and DMS that track learning, suggesting both structures contribute to the ability of humans to learn new alternative, memory-dependent responses (Brown and Stern, 2014). Looking forward, an especially open area for future imaging research is testing whether different learning dynamics which have been observed in rodent dorsal striatal subdivisions (Thorn et al., 2010) underlie development of model-based and model-free navigational behavior in humans. An important recent discovery suggests that even a distinction between DMS and DLS for comparatively flexible and inflexible navigation may be insufficient (Ferbinteanu, 2016). They found that the contributions of dorsal striatal subdivisions to memory-guided behavior are also influenced by training history. DMS was found to support both response-based and spatial (hippocampal-dependent) navigational strategies, consistent with a role for this region in translating mnemonic content into ongoing behavior. However, they also found that the DLS and hippocampus could support their respective alternative place- and response-based navigational strategies if the animal was concurrently trained to solve the task based on both types of cues. This work suggests that the entirety of the dorsal striatum can contribute, in some circumstances (see also Miyoshi et al., 2012), to navigational behaviors which also draw upon spatial mapping mechanisms.

## Future Directions

The data reviewed above suggest that a particularly fruitful direction for navigation research is systematic examination of: (1) the contributions of different striatal subdivisions in navigation under different learning conditions; and (2) how interactions between these subregions with spatial and non-spatial contextual information from the hippocampus (putatively mediated via the PFC and VS) drives flexible decision-making behavior. Prior work juxtaposing reward, action, and route representations in VS, dorsal striatum and hippocampus (van der Meer et al., 2010) sets the stage for examining differences within dorsal striatal subregions in a similar manner.

Another critical direction for future work converges with active research into the hierarchical organization of the PFC (Desrochers and Badre, 2012). Given evidence that dorsal striatal function may be organized according to its reciprocal connections with prefrontal subdivisions (Haber et al., 2006; Haber and Knutson, 2010), future work should target striatal contributions to navigation through the lens of associated prefrontal functional subdivisions, with attention to how these hierarchies may enable us to juggle “habitual” impulses to landmark cues with contextual guidance from the declarative memory system. Studies targeting how networks incorporating the hippocampus and subdivisions along the ventromedial-dorsolateral extent of the striatum learn, detect and implement shifts from more model-based to model-free action dynamically as control demands change (Jiang et al., 2015; Chiu et al., 2017) will be of substantial impact. Future imaging research could also more explicitly focus on how “value” is assigned to locations, particularly from a reinforcement learning perspective (Simon and Daw, 2011; Khamassi and Humphries, 2012). Such work could advance our understanding behavioral flexibility in navigation, and potentially inform interventions that leverage incentives to improve learning in rehabilitation settings.

## Author Contributions

SG, JS and TB wrote and reviewed all aspects of the manuscript. SG generated the figure.

## Conflict of Interest Statement

The authors declare that the research was conducted in the absence of any commercial or financial relationships that could be construed as a potential conflict of interest.
